# Association of 17q12-q21 Asthma Risk Locus with Clinical Severity of Infant Respiratory Syncytial Virus Infection

**DOI:** 10.3390/biom15081056

**Published:** 2025-07-22

**Authors:** Kedir N. Turi, Christopher McKennan, Christian Rosas-Salazar, Tebeb Gebretsedik, Dawn C. Newcomb, Emma E. Thompson, James Gern, James Chappell, Larry Anderson, Carole Ober, Tina Hartert

**Affiliations:** 1 Department of Epidemiology and Biostatistics, Indiana University, 1025 E Seventh St., Bloomington, IN 47405, USA; 2 Department of Statistics, University of Pittsburgh, Pittsburgh, PA 15213, USA; chm195@pitt.edu; 3 Department of Pediatrics, Vanderbilt University Medical Center, Nashville, TN 37232, USA; c.rosas.salazar@vumc.org (C.R.-S.); jim.chappell@vumc.org (J.C.); tina.hartert@vumc.org (T.H.); 4 Department of Biostatistics, Vanderbilt University Medical Center, Nashville, TN 37203, USA; tebeb.gebretsadik@vumc.org; 5 Department of Medicine, Vanderbilt University Medical Center, Nashville, TN 37232, USA; dawn.newcomb@vumc.org; 6 Department of Human Genetics, University of Chicago, Chicago, IL 60637, USA; eethomps@uchicago.edu (E.E.T.); c-ober@bsd.uchicago.edu (C.O.); 7 Department of Pediatrics, University of Wisconsin, Madison, WI 53705, USA; gern@medicine.wisc.edu; 8 Department of Pediatrics, Emory University, Atlanta, GA 30322, USA; larry.anderson@emory.edu

**Keywords:** 17q12-q21-locus, respiratory syncytial virus (RSV), viral load, infant, infection, asthma

## Abstract

This study examined whether SNPs at the 17q12-q21 locus that are associated with childhood asthma are also associated with severe respiratory syncytial virus (RSV) infection and viral load. We conducted a candidate SNP association study in the subset of RSV-infected infants who were parent-identified as White (*n* = 159) in the INSPIRE cohort. Nine SNPs at the 17q12-q21 locus were genotyped. We used an additive model to evaluate each SNP’s association with RSV infection severity and viral load. Replication of significant associations was tested in the TCRI cohort: infants with severe RSV illness. In INSPIRE, an SNP rs8069202-G in the *GSDMA* gene was associated with increased RSV viral load (and marginally associated with RSV severity). SNP rs2941504, in the *PGAP3* gene, was associated with a reduced risk of RSV severity. All significant associations were directionally replicated in the TCRI cohort but were insignificant at a *p*-value < 0.05. The association of a SNP in *GSDMA* with RSV viral load and RSV infection severity suggests that *GSDMA* may be contributing to both severe RSV infection and asthma development. On the other hand, the association between an SNP in *PGAP3* and reduced RSV infection severity suggests distinct pathways link *PGAP3* to these two respiratory outcomes.

## 1. Introduction

Early life respiratory syncytial virus (RSV) lower respiratory tract infections (LRTIs) have been associated with the risk of childhood asthma [1,2,3]. Whether severe RSV infection early in life causes asthma or simply identifies infants who are genetically predisposed to develop subsequent wheezing and asthma has long been debated. We previously demonstrated a dose–response relationship between RSV infection severity and asthma risk [4], as well as the likely contribution of family history to both conditions [5,6]. Thus, understanding the contribution of genetics to both conditions is important in advancing our understanding of the association between RSV LRTI and asthma, as well as in identifying the genetic underpinnings of enhanced susceptibility to respiratory viral infections among asthmatics.

The fact that not all infants who are infected with RSV develop lower respiratory tract infection (LRTI) and not all infants with RSV LRTI develop childhood asthma suggests that complex interactions between the child’s genetic makeup and other in utero and early life environmental exposures are important determinants of RSV infection severity and asthma development [7]. Numerous GWAS and meta-analyses of GWAS have identified and consistently replicated strong associations between genetic variants at a locus on chromosome 17q12-q21 and childhood-onset asthma [8,9,10,11]. Studies show that carriers of the asthma-associated risk allele at 17q21 exhibit increased expression of the corresponding SNP, especially in genes such as *GSDMB* and *ORMDL3* [12,13,14]. Childhood-onset asthma [8] is often manifested during virally triggered disease exacerbations [15,16,17]. Further, recent studies demonstrate that the 17q21 asthma-risk alleles and early-life viral infections synergistically promote airway inflammation in pediatric patients [18,19]. However, understanding the pathological mechanism that links 17q12-q21 region variants with asthma pathogenesis has been challenging because the region is comprised of 10 candidate genes in strong linkage disequilibrium in European populations. SNPs at this locus are also associated with the expression of asthma-related and immune response genes in this region [20,21], thereby making them key candidates for clinical biomarkers in asthma pathogenesis and possibly viral infection severity.

The objectives of this study were to investigate whether alleles previously known to be associated with childhood asthma risk at the 17q12-q21 locus are also associated with severe RSV infection and viral load.

## 2. Methods

### 2.1. Study Design

We conducted a candidate SNP association study to investigate the relationship between alleles at the 17q12-q21 locus and both RSV infection severity and viral load during acute RSV infection during infancy.

### 2.2. Population

The study sample was drawn from the INSPIRE (Infant Susceptibility to Pulmonary Infections and Asthma Following RSV Exposure) birth cohort, designed to study the role of infant (first year of life) RSV infection on later respiratory outcomes. INSPIRE is a population-based cohort of term normal birth weight healthy infants recruited from the central Tennessee region in the United States, which includes rural, suburban, and urban areas. The cohort recruitment, survey assessments, and biospecimen collection methods have been previously described [22]. Among 1949 INSPIRE cohort infants, 16% (*n* = 320) met the criteria for an acute respiratory illness (ARI) and had RSV confirmed by PCR. We excluded those with coinfections. The current analysis included genotypes for 17q12-q21 SNPs that were available for the subset who agreed to the use of their DNA, had infant RSV ARI (*n* = 229), and were parent-identified as White (*n* = 159). We focused on this group because both the allele frequencies at 17q12-q21 SNPs [8] and RSV infection frequencies [23,24] and asthma incidence [25,26] differ between racial and ethnic groups and because there were too few infants who were parent-identified as Black (*n* = 31), Hispanic (*n* = 19), or other/multi-race/unknown (*n* = 16) in the study sample. Table 1 provides the demographic and other characteristics of the infants included in this analysis.

The Tennessee Children’s Respiratory Initiative (TCRI) served as a replication cohort. The TCRI cohort enrolled normal birth weight and otherwise healthy infants at the time of an acute respiratory illness visit (hospitalization, emergency department, or unscheduled outpatient visit) for presumed respiratory tract infection during four (2004–2008) winter virus seasons [27]. A subset of TCRI infants who parent-identified as White, with RSV infection, who were genotyped, and who had PCR-based measurements of RSV abundance were used for replication studies (*n* = 168). Table S1 provides demographic and other covariate characteristics of the TCRI replication cohort.

One parent of each participant provided written informed consent for participation in the INSPIRE and TCRI studies. The protocol and informed consent documents were approved by the institutional review board of Vanderbilt University Medical Center (IRB approval for INSPIRE [IRB number 111299] and TCRI [IRB number 040849]).

### 2.3. Acute Respiratory Illness Visits and Nasal Wash Sample Collection (INSPIRE)

Infants underwent biweekly surveillance for ARI during the winter virus season (November through March). If infants met the predefined criteria for an ARI, a respiratory illness visit was conducted to assess the disease severity, and a nasal wash sample was collected [22]. The nasal wash was performed using 5 mL of sterile saline, immediately placed at 4 °C for transport, and then snap frozen at −80 °C for storage.

### 2.4. Acute Respiratory Infection Severity (INSPIRE)

Two measures of RSV ARI severity were used: an ordinal respiratory severity score and a dichotomous classification of upper respiratory tract infection (URTI) and lower respiratory tract infection (LRTI). The respiratory severity score of the RSV ARI was determined by a brief physical exam including the following measures: respiratory rate, oxygen saturation, heart rate, wheezing, and chest retractions. Chart review was used to supplement the clinical research exam with information about physician diagnosis and when a healthcare visit occurred. The respiratory symptom assessment detailed the symptoms present and the date of onset. The ordinal respiratory severity score was calculated to assess infection severity (scale 0–12), with higher values indicating a more severe clinical illness [28]. The respiratory severity score distinguishes disease severity based on URTI and LRTI. Additionally, the ARI was classified into upper or lower respiratory tract infection (URTI and LRTI), respectively.

### 2.5. Respiratory Viral Detection and Viral Load (INSPIRE)

Nasal samples collected during an ARI were profiled for respiratory viruses (RSV and human rhinovirus [HRV]) that cause the majority of infant respiratory viral illness. Virus detection and viral load were determined using reverse-transcription real-time polymerase chain reaction (PCR) [29]. The PCR cycle threshold (Ct) served as a proxy for the viral load.

### 2.6. Genotyping (INSPIRE and TCRI)

Genotyping for a pre-selected set of SNPs at the 17q12-q21 locus was performed at the University of Chicago using 500 ng of DNA at a concentration of 5 ng/μL. Nine SNPs were selected based on linkage disequilibrium patterns at 17q12-q21 and their association with childhood asthma in previous GWAS studies or with the expression of 17q12-q21 genes [8]. The genotyping methods and quality control have been previously described [30]. Genotypes were coded as 0 (genotype with 0 asthma risk-associated alleles), 1 (genotype with 1 asthma risk-associated allele), and 2 (genotype with 2 asthma risk-associated alleles) for additive models, where the asthma risk-associated allele was defined according to Stein et al. [8] and Ober et al. [30].

### 2.7. Statistical Analysis

The associations between SNP genotypes and the continuous respiratory severity score as one indicator of RSV infection severity were assessed using linear regression, and the relationships between genotypes and the dichotomous classification of respiratory infection severity (LRTI vs. URTI) were assessed using logistic regression. Associations between the 17q12-q21 SNPs and RSV infection severity score were assessed using linear regression. The associations between the SNP genotypes and viral load (inverse of PCR Ct values) were assessed using linear regression. We used genotype data from INSPIRE infants who parent-identified as White to estimate the linkage disequilibrium (LD) between 17q12-q21 SNPs. Considering the strong linkage disequilibrium between 17q12-q21 SNPs (r^2^ > 0.80), the nine SNPs in 17q12-q21 represented three independent loci, and therefore, we used a multiple testing-corrected *p*-value threshold of 0.05/3 = 0.02. All models were adjusted for infant age at ARI, number of days of symptoms at the time of sample collection, sex, secondhand smoke exposure, and breastfeeding. Replication analysis was conducted in the TCRI cohort for significant association results from the INSPIRE cohort analysis, adjusting for the same covariates. We used a one-tailed *p*-value for statistical significance in the replication analysis. R software version 4.5.1 was used for data processing and statistical analysis. 

### 2.8. Integrating Previously Published Single-Cell and Bulk Gene Expression Data for Significant Genes

For SNPs significantly associated with RSV severity or viral load, we analyzed publicly available single-cell and gene expression data from LungMAP [31,32]. Specifically, we examined whether the genes linked to these SNPs were expressed in immune and airway cells relevant to the pathways through which the genes exert their effects.

In addition, given the lack of publicly available RSV gene expression data, evidence from multiple HRV infection studies was used to assess potential differential gene expression involving the SNPs significantly associated with RSV infection severity [33]. The gene expression data included in this analysis include multiple published studies that used heterogeneous tissues such as cytotoxic T-lymphocyte, peripheral blood mononuclear cell, nasal mucosa, bronchial epithelial cell, peripheral blood, and tracheobronchial epithelial cell [33].

## 3. Results

### 3.1. Demographics of the Study Population

The demographics of the infant study population with RSV infection are detailed in Table 1 (INSPIRE cohort) and Table S1 (TCRI cohort). In the INSPIRE testing cohort, there were 159 infants who parent-identified as White with a first RSV infection, for whom genotype data were available, among whom 68 (41.5%) had an RSV-LRTI (Table 1). In the replication cohort, TCRI, there were 168 infants who parent-identified as White with genotype data, and almost all (97%) (*n* = 163) had LRTI, which is not unexpected given this was a study designed to enroll previously healthy infants with acute respiratory illness requiring acute or hospital-level care (Table S1). See supplemental text for a detailed description of both INSPIRE and TCRI sub-populations.

### 3.2. Association of 17q12-q21 Locus SNPs and Infant RSV Infection Severity

**Figure 1:**
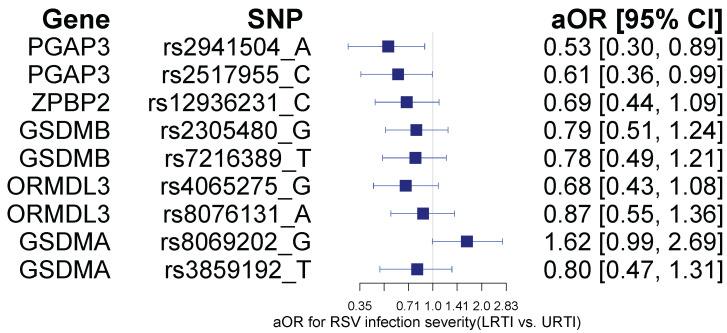
Forest plot of the association between 17q12-q21 locus selected SNPs and respiratory syncytial virus lower respiratory tract infection (RSV LRTI). Regressions were adjusted for sex, infant age at illness, days since illness onset, breastfeeding status, and secondhand smoke exposure.

Two of nine SNPs (rs2517955 and rs2941504) were nominally associated with RSV LRTI before adjusting for multiple testing, but only rs2941504 was significant after adjusting for multiple testing (Figure 1). The two SNPs (rs2517955 and rs2941504) are in high LD (r^2^ = 0.80) and located in the *PGAP3* gene region. Alleles rs2517955-C (adjusted odds ratio, aOR = 0.61; 95% confidence interval (CI) = [0.36, 0.99], *p* = 0.049) and rs2941504-A (aOR = 0.61; 95% CI = [0.36, 0.99], *p* = 0.017) were inversely associated with RSV LRTI diagnosis. On the other hand, rs8069202-G near the Gasdermin A gene (*GSDMA*) was nominally associated with LRTI (aOR = 1.62 [95% CI = 0.99, 2.69], *p* = 0.059. We did not find significant associations between the 17q12-q21 SNPs and RSV infection severity score including rs2941504 (β = 0.18; 95% CI = [−0.22, 0.58], *p* = 0.37) and rs2517955 (β = 0.08; 95% CI = [−0.31, 0.47], *p* = 0.68). The distribution of SNP genotypes by RSV LRTI (vs. URTI) is shown in Table S2.

### 3.3. Association of 17q12–21 Locus SNPs and RSV Viral Load

**Figure 2:**
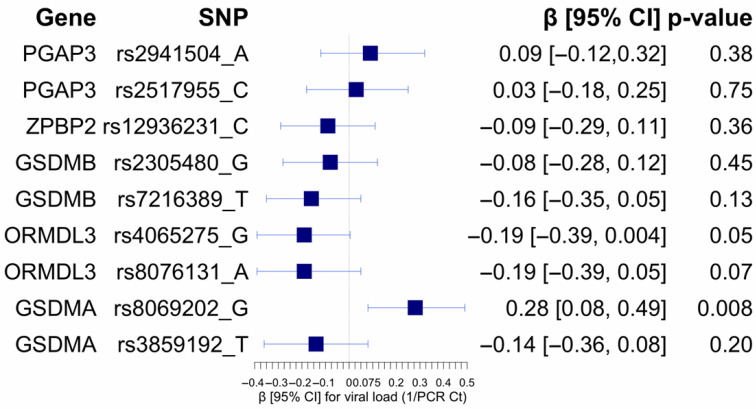
Forest plot of the association between 17q12-q21 locus selected SNPs and viral load (PCR-Ct). Regressions were adjusted for sex, infant age at illness, days since illness onset, breastfeeding status, and secondhand smoke exposure.

The allele rs8069202-G near the GSDMA gene region was associated with increased viral load (β = 0.28; 95% CI = [0.08, 0.49]; *p* = 0.008) (Figure 2).

### 3.4. Replication of Results in the TCRI Cohort

We conducted a replication analysis for significantly associated results from the INSPIRE cohort (Table S3). The direction of the associations was the same between rs2941504 (aOR = 0.82; 95% CI = [0.45, 1.52], P_α/2_ = 0.27), rs2517955 (aOR = 0.94; 95% CI = [0.52, 1.70], P_α/2_ = 0.43), and RSV LRTI (vs. URTI), as in INSPIRE, but these were not statistically significant. The associations between rs2941504 (β = 0.27; 95% CI = [−0.38, 0.73], P_α/2_ = 0.23) and rs2517955 (β = −0.29; 95% CI = [0.37, 0.77], P_α/2_ = 0.22) and the RSV severity score were not statistically significant. The direction of the association was the same between rs8069202 and RSV viral load measured by the inverse PCR Ct (β = 0.0009; 95% CI = [−0.0002, 0.002], P_α/2_ = 0.05), as in the INSPIRE cohort but was not statistically significant (see Table S4 for genotype distribution of the selected SNPs by RSV severity category).

### 3.5. LungMAP Single-Cell Data and Differential Gene Expression Data of PGAP3 and GSMDA

**Figure 3:**
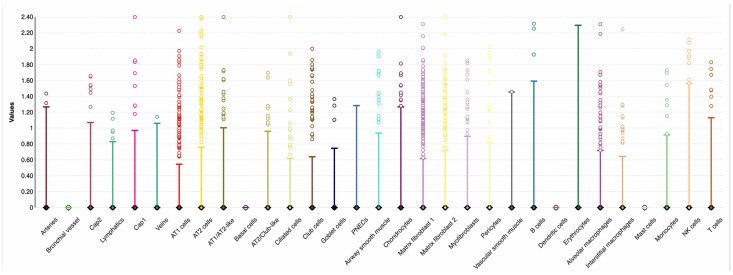
LungMAP single-cell mRNA data showing *PGAP3* expression by lung cell types in a 31-week-old human infant. The *x*-axis shows cell types, and the *y*-axis shows normalized expression. CAP = cyclase-associated protein, NK = natural killer, PNECs = pulmonary neuroendocrine cells, and AT = pulmonary alveolar type.

**Figure 4:**
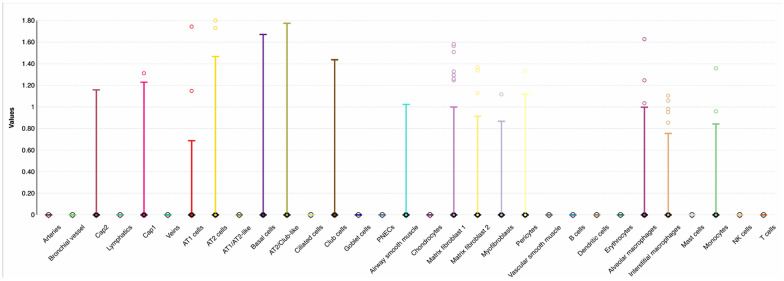
LungMAP single-cell mRNA data showing the *GSDMA* expression by lung cell types in a 31-week-old human infant. The *x*-axis shows cell types, and the *y*-axis shows normalized expression. CAP = cyclase-associated protein, NK = natural killer, PNECs = pulmonary neuroendocrine cells, and AT = pulmonary alveolar type.

For the SNPs significantly associated with RSV LRTI (rs2941504) and viral load (rs8069202), we searched publicly available LungMAP (lung gene expression analysis) [31,32] single-cell data to determine whether genes of these SNPs were expressed in lung and immune cells. The single-cell data available in LungMAP are from a 31-week-old human infant with no lung disease. Accordingly, *PGAP3* (SNP rs2941504) was highly expressed in innate and adaptive immune cell types relevant to abating viral infection as B cell, natural killer (NK) cells, T cells, monocytes, and alveolar and interstitial macrophages (Figure 3). *GSDMA* (SNP rs8069202) was expressed mainly in epithelial cells (club cells and alveoli-type cells), innate immune cell types such as macrophages (alveolar and interstitial), and monocytes (Figure 4). However, we should note that the data are not related to RSV infection and are only meant to show that these genes are expressed in respiratory infection-relevant tissue and cells.

**Figure 5:**
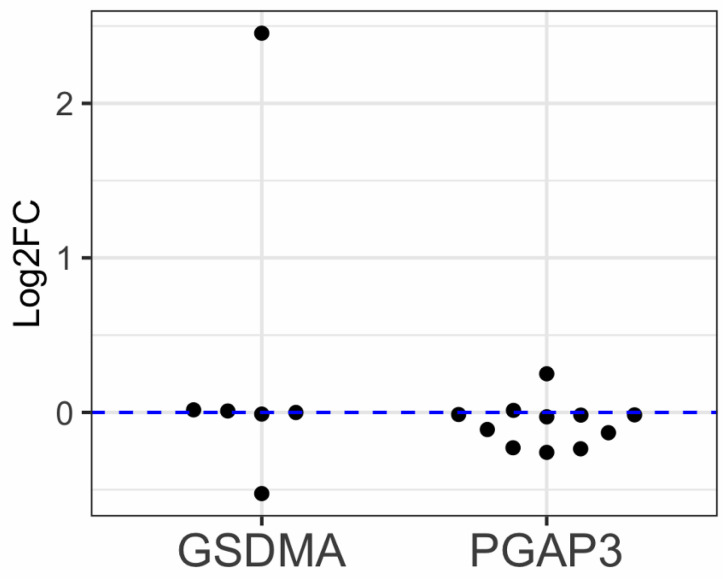
Differential gene expression of *GSDMA* and *PGAP3* during human rhinovirus (HRV) infection across multiple studies. The *x*-axis shows genes, and the *y*-axis shows log2 fold change of the studies. Each dot represents a study.

In the absence of publicly available RSV gene expression data, we further supported our hypothesis by referencing findings from multiple studies that demonstrated an increase in the gene expression of *GSDMA* and a decrease in the gene expression of *PGAP3* following HRV infection (Figure 5). Summary results from several studies show a mean log2 fold change of 0.32 for *GSDMA* (*n* = 6) and a mean log2 fold change of −0.07 for *PGAP3* due to HRV A infection. Although these findings do not constitute direct evidence for changes due to RSV, they suggest the possibility of a similar gene expression response that could occur following RSV infection.

## 4. Discussion

This study tested the hypothesis that SNPs at the 17q12-q21 asthma risk locus would be associated with RSV infection severity in infants. Our hypothesis was driven by the well-established association between severe RSV and asthma risk, an association that is confounded by a family history of asthma and could therefore be due to shared genetics. Given that 17q12-q21 is the strongest and most replicated locus associated with childhood-onset wheeze and asthma, we sought to test the association between alleles at this asthma risk locus and the severity of infant RSV infection [1,7,8]. Contrary to our hypothesis, in this study we found that seven of nine SNPs at the 17q12-q21 asthma risk locus were not associated with RSV infection severity. One SNP (rs8069202) was associated with increased RSV viral load and nominally associated with RSV severity as measured by LRTI, and one SNP (rs2941504) was associated with decreased risk, rather than our hypothesized increased risk of severe infant RSV infection. At both SNPs, the associated alleles (G and A, respectively) occur on the asthma-associated haplotype in Europeans.

**Figure 6:**
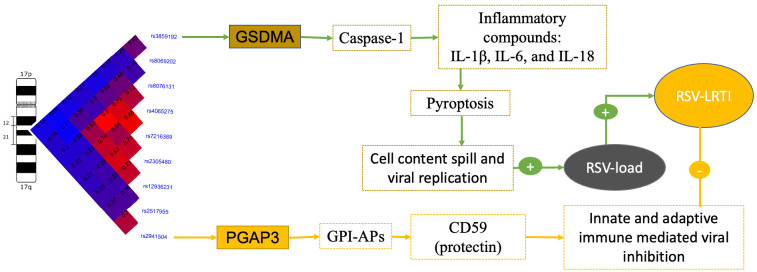
Summary of the results of the association between SNPs in the 17q12-q21 locus and RSV infection severity and viral load. The hypothesized pathways of the effect from SNPs to the clinical and viral load outcomes are consistently colored (i.e., boxes and lines connecting each SNP to outcomes have the same color for each SNP). The plus and minus signs represent the directions of the associations of the SNPs and outcomes.

To provide new insights into the potential molecular pathways of the effect of asthma risk locus SNPs on the pathogenesis of early-life severe RSV infection, we synthesized the published literature on the function of genes of SNPs significantly associated with RSV severity in our study. The speculated pathways between asthma risk locus alleles and severe RSV infection are summarized in Figure 6. First, the association between allele rs2941504-A in *PGAP3* (previously associated with asthma risk) [8,34] and decreased risk of RSV LRTI led us to speculate the involvement of *PGAP3* in distinct downstream mechanisms of asthma development and RSV LRTI risks. We can speculate on two plausible explanations for this unexpected association of alleles in *PGAP3* and protection from RSV LRTI. Given that rs2941504 resides within a *PGAP3* intron, other variants in the gene may contribute to differing associations with asthma and LRTI by influencing alternative splicing and diversifying gene function [34]. It has been demonstrated that SNPs in LD with rs2941504 form a functional haplotype that influences alternative splicing of *PGAP3* and multiple transcript variants encoding distinct isoforms [35]. In addition, *PGAP3*-regulated glycosylphosphatidylinositol (GPI) anchors [36] and GPI-anchored proteins (GPI-APs) are known to play roles in the immune responses, including the complement cascade, macrophage activation, and T cell proliferation [37,38,39,40,41]. Additionally, a specific haplotype at SNP rs2941504 has been associated with significantly increased levels of several soluble GPI-Aps in those with asthma [35,37,42,43]. Based on these findings, we propose that the protective effect against severe RSV infection may be mediated through immune signaling pathways driven by soluble GPI-Aps. Further, viruses with a capsid, such as RSV, are susceptible to inactivation by complement [44,45,46], suggesting additional indirect mechanisms of *PGAP3* polymorphisms attenuating the severity of RSV infection. Whether alternative splicing or GPI-Aps-driven immune signaling pathways underlie the divergent association of *PGAP3* with asthma risk and RSV LRTI is a topic for future research.

The second finding of our study was that infants with the rs8069202_G in *GSDMA* had higher RSV viral loads during acute upper airway infection, and there was a non-significant trend with the risk of RSV LRTI. This observation suggests that impaired host control of viral replication may lead to either enhanced clinical illness severity or viral persistence [47,48,49]. Evidence supporting this has been demonstrated in vitro in asthmatic airway epithelial cells [50] and in vivo in innate lymphoid cells [51] in the context of human rhinovirus infection. While *GSDMA* is plausibly involved in asthma pathogenesis through immune inflammasome [52,53] and mitochondrial reactive oxygen species generation [54] pathways, it is also a precursor of pore-forming proteins and acts as a sensor of infection [55,56]. The gasdermin family of genes promotes pyroptosis in response to bacterial and viral infections, sterile danger signals, or cytotoxic T cell attacks [57,58]. Although not genotype-specific, the expression level of another gasdermin family gene (*GSDMB*) has been shown to significantly increase in HRV-stimulated PBMCs, as compared with unstimulated PBMCs [15]. Further, single-cell gene expression data from LungMAP [32] show that *GSDMA* is expressed in lung macrophages and monocytes, providing many potential pathways by which *GSDMA* might regulate host response to RSV infection. RSV-infected macrophages induce both pyroptosis and necroptosis, where pyroptosis is mediated by caspase-1 following activation of the ASC-NLRP3 inflammasome [59], although it is not yet known under what circumstances the switch from apoptosis to pyroptosis or necroptosis occurs. Pyroptosis increases viral shedding and replication in some infections [60], which is consistent with our finding that rs8069202-G was positively associated with increased RSV viral load. Taken together, it is reasonable to speculate that rs8069202-G in the *GSDMA* increases viral replication through immune inflammasome-led pyroptosis and mitochondrial reactive oxygen species (ROS) generation, leading to enhanced RSV infection severity. Further investigation is needed to determine whether the same *GSDMA*-mediated immune inflammasome and ROS pathways are involved in both childhood asthma development and RSV infection severity. As we did not see an association with the causal asthma SNP rs2304580 or at least one of the SNPs in *GSDMB* or *ORMDL3*, it suggests that the genetic risk at this locus for both asthma and for RSV infection (severity and viral load) are different. It may be that different genes are important for asthma (*GSDMB* or *ORMDL3*) and severe RSV infection (*GSDMA* or *PGAP3*); however, because of the small sample size for this candidate gene study and lack of replication, this hypothesis requires additional study to understand these associations.

Our study has several notable strengths. It addresses a novel and important question by examining the association between asthma risk alleles and RSV infection severity. The study was conducted in a well-characterized population-based birth cohort of term healthy infants, specifically designed to capture the first infant RSV ARI. Additionally, the study utilized objective measures of infection severity and included measurement of viral load, further enhancing the rigor of the design. Despite these strengths, several limitations must be considered. The first and main limitation of this study is the small sample size for a candidate SNP study. However, the number of SNPs a priori selected for this study is small, and most of them are in LD with each other, thus limiting the number of independent tests. Second, even though our cohort is population-based with race and ethnicity representation similar to the general population from which the sample was drawn, due to the small sample size, we were only able to consider infants who were parent-identified as White for this analysis. Since both genetic structure (allele frequency and LD) at 17q12-q21 and severity of RSV infection differ by race and ethnicity, this is missed opportunity that might have explained the RSV infection severity disparity between these groups. Third, while we conducted a replication analysis in an independent cohort, and the direction of the significant results were the same, the results were not significant in the replication cohort. The non-replication result could be due to the difference between the discovery (INSPIRE) and replication (TCRI) cohorts, where INSPIRE enrolled healthy infants (birth cohort healthy at enrollment) and TCRI enrolled infants hospitalized due to respiratory illness. Fourth, even though we ascertained the number of reported days from ARI symptom onset to sample collection, it was not possible to know the exact number of days from actual viral inoculation to the nasal sampling date in the assessment of viral load (Ct values). To address this, we adjusted for the number of days from symptom onset to the nasal sample collection, but we recognize that this is an imperfect measure of the time from infection to sampling. Fifth, we did not examine the relationship between the SNPs and asthma outcome and immune response in this study. However, several studies, including large-scale meta-analyses, have demonstrated that the 17q21 locus is robustly associated with asthma [8,9,10,11].

## 5. Conclusions

Our study identified an association between SNP (rs8069202) at the 17q12-q21 asthma risk locus, near the *GSDMA* gene, and increased RSV viral load, as well as a non-significant trend with increased RSV LRTI risk. This association may be explained by inflammasome-led pyroptosis and reactive oxygen species pathways, which are shared mechanisms involved in asthma development and may also explain the increased asthma morbidity observed with respiratory viral infection. Conversely, the inverse association between rs2941504 in *PGAP3* and RSV LRTI severity suggests that pathways involving GPI-APs regulated by *PGAP3* gene region and known to skew immune responses toward a T2 profile characteristic of atopic asthma may confer protection from severe viral infections. In summary, the contrasting associations of SNPs in *PGAP3* with reduced RSV infection severity suggest distinct pathways linking *PGAP3* to these respiratory outcomes. However, given the limited sample size, further studies are needed to clarify and better understand these relationships.

## Figures and Tables

**Table 1:** Infant characteristics by RSV respiratory illness severity (upper respiratory tract infection [URTI] and lower respiratory tract infection [LRTI]). The study sample from INSPIRE cohort was limited to RSV-positive participants who parent-identified as White and with genotype data available.

	RSV LRTI (*n* = 68)	RSV URTI (*n* = 91)	Missing (*n* = 4)	Total (*n* = 163)	*p*-Value *
Sex					0.81
Male	35 (51.5%)	51 (56.0%)	2 (50.0%)	89 (54.3%)	
Female	33 (48.5%)	40 (44.0%)	2 (50.0%)	75 (45.7%)	
Days since illness onset (days)					0.34
Mean (SD)	3.48 (1.68)	3.10 (1.56)	3.50 (1.00)	3.27 (1.60)	
Median [Min, Max]	3.00 [0, 7.00]	3.00 [0, 7.00]	4.00 [2.00, 4.00]	3.00 [0, 7.00]	
Missing	1 (1.5%)	0 (0%)	0 (0%)	1 (0.6%)	
Age at illness (Months)					0.45
Mean (SD)	4.01 (1.84)	4.29 (2.16)	NA (NA)	4.16 (2.03)	
Median [Min, Max]	4.00 [0, 8.00]	4.00 [0, 9.00]	NA [NA, NA]	4.00 [0, 9.00]	
Missing	0 (0%)	0 (0%)	4 (100%)	4 (2.4%)	
Viral load (RSV PCR Ct)					0.05
Mean (SD)	24.1 (4.77)	24.9 (5.30)	30.4 (6.54)	24.7 (5.16)	
Median [Min, Max]	23.3 [14.6, 38.0]	24.5 [16.4, 37.6]	30.8 [22.9, 37.0]	24.0 [14.6, 38.0]	
RSV Illness Severity Score					
Mean (SD)	3.71 (1.82)	2.28 (1.25)	NA (NA)	2.89 (1.67)	<0.001
Median [Min, Max]	3.00 [1.00, 10.0]	2.00 [0, 6.00]	NA [NA, NA]	3.00 [0, 10.0]	
Missing	3 (4.4%)	4 (4.4%)	4 (100%)	11 (6.7%)	
Secondhand smoke exposure					0.19
Exposed	34 (50.0%)	34 (37.4%)	1 (25.0%)	69 (42.1%)	
Not exposed	34 (50.0%)	57 (62.6%)	3 (75.0%)	95 (57.9%)	
Any breastfeeding					0.20
Yes	49 (72.1%)	76 (83.5%)	3 (75.0%)	129 (78.7%)	
No	19 (27.9%)	15 (16.5%)	1 (25.0%)	35 (21.3%)	

* *p*-value was calculated using ANOVA test for continuous variables and chi-square for categorical variables. NA = not available.

## Data Availability

Genetic data were generated at the University of Chicago. Cohort data supporting the findings of this study are available from the senior author [TVH] on request.

## Supplementary Materials

The following supporting information can be downloaded at https://www.mdpi.com/article/10.3390/biom15081056/s1, Supplemental Result Text S1: Results; supplemental Figure S1: Linkage disequilibrium (r^2^) matrix between selected 17q12-q21 locus selected SNPs from a subset of INSPIRE cohort children who parent-identified as White; Supplemental Table S1: TCRI study participant characteristics by respiratory illness severity (upper respiratory tract infection [URTI] and lower respiratory tract infection [LRTI]). The study was limited to RSV-positive participants who parent-identified as White and with available genotype data; Table S2: SNPs in the 17q12-q21 locus by RSV infection severity (upper respiratory tract infection [URTI] and lower respiratory tract infection [LRTI]) among those who parent-identified as White in the INSPIRE cohort; Table S3: Association between 17q12-q21 locus and RSV severity (upper respiratory tract infection [URTI] vs. lower respiratory tract infection [LRTI]), and viral load in the TCRI cohort (replication cohort). Regressions were adjusted for sex, infant age at illness, breastfeeding status, and secondhand smoke exposure; Table S4: TCRI study (replication cohort) sample 17q12-q21 locus selected SNP genotype distribution by RSV infection severity (upper respiratory tract infection [URTI] and lower respiratory tract infection [LRTI]) among study participants who parent-identified as White.
