# Supplementary material for: Association of 17q12-q21 Asthma Risk Locus with Clinical Severity of Infant Respiratory Syncytial Virus Infection
Source: Biomolecules. 2025 Jul 22;15(8):1056. doi: 10.3390/biom15081056 (PMC12383441; doi:10.3390/biom15081056)
Supplement: Supplementary file 1 [file biomolecules-15-01056-s001.zip › biomolecules-3689952-supplementary.pdf]

Supplemental Material

Title: Association of 17q12-q21 Asthma Risk Locus with Clinical Severity of Infant Respiratory Syncytial Virus Infection

Kedir N Turi, Christopher McKennan, Christian Rosas-Salazar, Tebeb Gebretsedik, Dawn C Newcomb, Emma E Thompson, James Gern, James Chappell, Larry Anderson, Carole Ober, Tina Hartert

Supplemental text

Supplemental Text S1. Results

17q12-q21 locus SNPs and RSV viral load

In the additive models of association between17q12-q21 locus SNPs and RSV viral load, SNP rs8069202-G (β=0.28; 95%CI=[0.08, 0.49]; p-value=0.008) was positively associated with increased viral load. The association between SNP rs8069202-G and viral load cleared the multiple testing burden ($p<0.017$). rs8069202 is in the *GSDMA* gene region.

In addition, SNPs rs4065275-G (β=-0.19; 95%CI=[-0.39, 0.04]; p-value=0.05) and SNP rs8076131-A (β=-0.19; 95%CI=[-0.39, 0.05]; p-value=0.07) were marginally inversely associated with increased viral load. The association between SNP rs8069202 and viral load cleared the multiple testing burden ($p<0.0125$) and the direction of the associations for the two marginally associated SNPs (rs8076131 and rs4065275) are opposite of SNP rs8069202. SNP rs8069202-G is in the *GSDMA* gene region; SNP rs8076131 and SNP rs4065275-G are in the *ORMDL3* gene region and are in LD (Figure S1).

Profile of replication sample (TCRI cohort subsample) by RSV severity outcome

In the TCRI subset who parent-identified as White and with available genotype data (n=207), 85% (n=177) had RSV LRTI. Please see the characteristics of the TCRI study participants in Table S1.

Supplemental Figure Legends

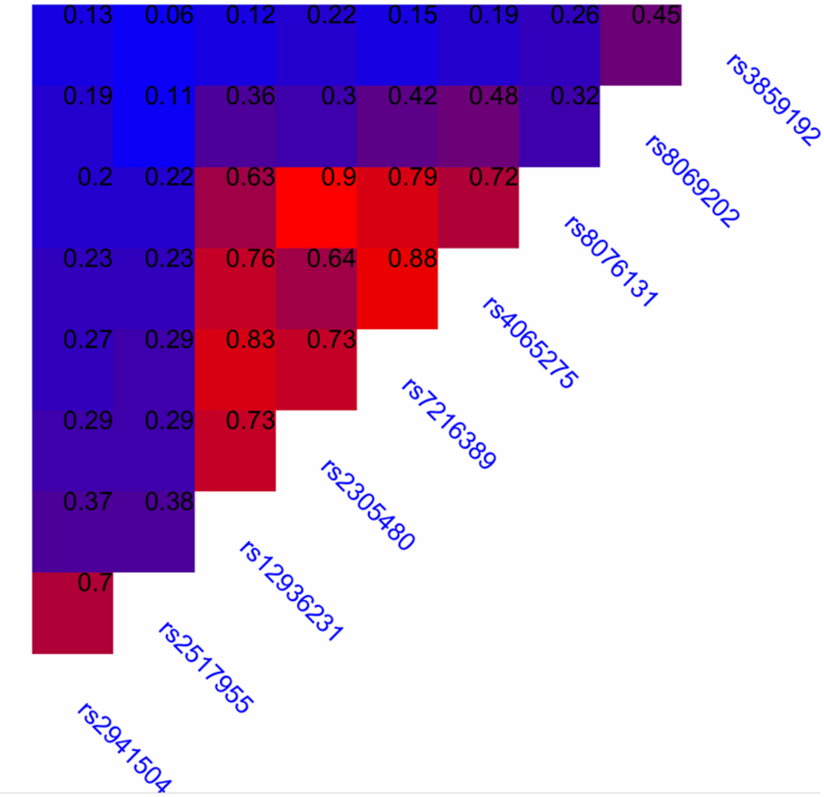

Figure S1. Linkage disequilibrium (r^2) matrix between selected 17q12-q21 locus selected SNPs from a subset of INSPIRE cohort children who parent-identified as White

Supplemental Tables

Table S1. TCRI study participant characteristics by respiratory illness severity (upper respiratory tract infection [URTI] and lower respiratory tract infection [LRTI]). The study was limited to RSV-positive participants who parent-identified as White and with available genotype data

	LRTI<br>**(N=167)**	URTI<br>**(N=28)**	Total<br>**(N=195)**
**Sex**			
Male	92 (55.1%)	17 (60.7%)	109 (55.9%)
Female	75 (44.9%)	11 (39.3%)	86 (44.1%)
**Days since illness onset (days)**			
Mean (SD)	6.19 (7.04)	5.19 (6.14)	6.06 (6.92)
Median [Min, Max]	4.00 [0, 62.0]	4.00 [-9.00, 21.0]	4.00 [-9.00, 62.0]
Missing	22 (13.2%)	7 (25.0%)	29 (14.9%)
**Age at illness (Weeks)**			
Mean (SD)	13.9 (11.6)	19.6 (14.6)	14.7 (12.2)
Median [Min, Max]	10.0 [2.00, 53.0]	14.0 [3.00, 51.0]	10.0 [2.00, 53.0]
**Viral load (RSV PCR Ct)**			
Mean (SD)	28.8 (3.89)	29.1 (7.22)	28.8 (4.00)
Median [Min, Max]	28.6 [19.8, 39.1]	25.3 [22.8, 39.8]	28.5 [19.8, 39.8]
Missing	25 (15.0%)	23 (82.1%)	48 (24.6%)
**Respiratory illness severity score**			
Mean (SD)	6.94 (2.81)	1.23 (1.64)	6.12 (3.34)
Median [Min, Max]	7.00 [1.00, 12.0]	1.00 [0, 7.50]	6.50 [0, 12.0]
**Secondhand smoke exposure**			
Yes	104 (62.3%)	17 (60.7%)	121 (62.1%)
No	63 (37.7%)	11 (39.3%)	74 (37.9%)
**Any breastfeeding**			
1	93 (55.7%)	19 (67.9%)	112 (57.4%)
0	74 (44.3%)	9 (32.1%)	83 (42.6%)

Table S2. SNPs in the 17q12-q21 locus by RSV infection severity (upper respiratory tract infection [URTI] and lower respiratory tract infection [LRTI]) among those who parent-identified as White in the INSPIRE cohort

	**RSV LRTI** (N=68)	**RSV URTI** (N=91)	**Total** (N=159)
**rs2305480**			
A/A	19 (27.9%)	20 (22.0%)	39 (24.5%)
G/A	33 (48.5%)	42 (46.2%)	75 (47.2%)
G/G	16 (23.5%)	29 (31.9%)	45 (28.3%)
**rs2517955**			
T/T	33 (48.5%)	37 (40.7%)	70 (44.0%)
T/C	31 (45.6%)	38 (41.8%)	69 (43.4%)
C/C	4 (5.9%)	16 (17.6%)	20 (12.6%)
**rs2941504**			
G/G	40 (58.8%)	43 (47.3%)	83 (52.2%)
G/A	26 (38.2%)	34 (37.4%)	60 (37.7%)
A/A	2 (2.9%)	14 (15.4%)	16 (10.1%)
**rs3859192**			
C/C	22 (32.4%)	23 (25.3%)	45 (28.3%)
C/T	36 (52.9%)	50 (54.9%)	86 (54.1%)
T/T	10 (14.7%)	18 (19.8%)	28 (17.6%)
**rs4065275**			
A/A	25 (36.8%)	23 (25.3%)	48 (30.2%)
G/A	31 (45.6%)	45 (49.5%)	76 (47.8%)
G/G	12 (17.6%)	23 (25.3%)	35 (22.0%)
**rs7216389**			
C/C	25 (36.8%)	27 (29.7%)	52 (32.7%)
T/C	31 (45.6%)	42 (46.2%)	73 (45.9%)
T/T	12 (17.6%)	22 (24.2%)	34 (21.4%)
**rs8069202**			
A/A	9 (13.2%)	17 (18.7%)	26 (16.4%)
G/A	31 (45.6%)	51 (56.0%)	82 (51.6%)
G/G	28 (41.2%)	23 (25.3%)	51 (32.1%)
**rs8076131**			

	**RSV LRTI** (N=68)	**RSV URTI** (N=91)	**Total** (N=159)
G/G	20 (29.4%)	21 (23.1%)	41 (25.8%)
A/G	32 (47.1%)	45 (49.5%)	77 (48.4%)
A/A	16 (23.5%)	25 (27.5%)	41 (25.8%)
**rs12936231**			
G/G	25 (36.8%)	26 (28.6%)	51 (32.1%)
C/G	33 (48.5%)	41 (45.1%)	74 (46.5%)
C/C	10 (14.7%)	24 (26.4%)	34 (21.4%)

Table S3. Association between 17q12-q21 locus and RSV severity (upper respiratory tract infection [URTI] vs. lower respiratory tract infection [LRTI]), and viral load in the TCRI cohort (replication cohort). Regressions were adjusted for sex, infant age at illness, breastfeeding status, and secondhand smoke exposure.

		**RSV LRTI vs URTI**	**Viral load (inverse PCR Ct)**
**SNP**	**Risk allele**	**Adjusted OR [95%CI]**	**Adjusted β [95%CI]**
rs2941504	A	0.82 [0.45, 1.52]<br>$P_{\alpha/2}$=0.27	
rs2517955	C	0.94 [0.52, 1.70]<br>$P_{\alpha/2}$=0.43	
rs8069202	G	1.03 [0.58, 1.82]<br>$P_{\alpha/2}$=0.46	0.0009 [-0.0002, 0.002]<br>$P_{\alpha/2}$=0.05

$P_{\alpha/2}$=indicates one-tail p-value for the directional hypothesis test.

Table S4. TCRI study (replication cohort) sample 17q12-q21 locus selected SNP genotype distribution by RSV infection severity (upper respiratory tract infection [URTI] and lower respiratory tract infection [LRTI]) among study participants who parent-identified as White.

	LRTI **(N=167)**	URTI **(N=28)**	Total **(N=195)**
**2305480**			
A/A	36 (21.6%)	4 (14.3%)	40 (20.5%)
G/A	79 (47.3%)	18 (64.3%)	97 (49.7%)
G/G	52 (31.1%)	6 (21.4%)	58 (29.7%)
**rs2517955**			
T/T	71 (42.5%)	11 (39.3%)	82 (42.1%)
T/C	73 (43.7%)	13 (46.4%)	86 (44.1%)
C/C	23 (13.8%)	4 (14.3%)	27 (13.8%)
**rs2941504**			
G/G	79 (47.3%)	12 (42.9%)	91 (46.7%)
G/A	72 (43.1%)	12 (42.9%)	84 (43.1%)
A/A	16 (9.6%)	4 (14.3%)	20 (10.3%)
**rs3859192**			
C/C	36 (21.6%)	6 (21.4%)	42 (21.5%)
C/T	97 (58.1%)	15 (53.6%)	112 (57.4%)
T/T	34 (20.4%)	7 (25.0%)	41 (21.0%)
**rs4065275**			
A/A	41 (24.6%)	5 (17.9%)	46 (23.6%)
G/A	80 (47.9%)	19 (67.9%)	99 (50.8%)
G/G	46 (27.5%)	4 (14.3%)	50 (25.6%)
**rs7216389**			
C/C	44 (26.3%)	5 (17.9%)	49 (25.1%)
T/C	77 (46.1%)	19 (67.9%)	96 (49.2%)
T/T	46 (27.5%)	4 (14.3%)	50 (25.6%)
**rs8069202**			
A/A	34 (20.4%)	4 (14.3%)	38 (19.5%)
G/A	84 (50.3%)	18 (64.3%)	102 (52.3%)
G/G	49 (29.3%)	6 (21.4%)	55 (28.2%)
**rs8076131**			

	LRTI **(N=167)**	URTI **(N=28)**	Total **(N=195)**
G/G	41 (24.6%)	4 (14.3%)	45 (23.1%)
A/G	76 (45.5%)	18 (64.3%)	94 (48.2%)
A/A	50 (29.9%)	6 (21.4%)	56 (28.7%)
**rs12936231**			
G/G	43 (25.7%)	5 (17.9%)	48 (24.6%)
C/G	80 (47.9%)	20 (71.4%)	100 (51.3%)
C/C	44 (26.3%)	3 (10.7%)	47 (24.1%)
